# Metabolomics study of osteopetrosis caused by *CLCN7* mutation reveals novel pathway and potential biomarkers

**DOI:** 10.3389/fendo.2024.1418932

**Published:** 2025-02-13

**Authors:** Xi Chen, Ziyuan Wang, Wenzhen Fu, Zhe Wei, Jiemei Gu, Chun Wang, Zhenlin Zhang, Xiangtian Yu, Weiwei Hu

**Affiliations:** ^1^ Shanghai Clinical Research Center of Bone Diseases, Department of Osteoporosis and Bone Diseases, Sixth People’s Hospital Affiliated to Shanghai Jiao Tong University School of Medicine, Shanghai, China; ^2^ Clinical Research Center, Sixth People’s Hospital Affiliated to Shanghai Jiao Tong University School of Medicine, Shanghai, China

**Keywords:** osteopetrosis, CLCN7 mutation, metabolomics, liquid chromatography-tandem mass spectrometry, glycerophospholipid metabolism

## Abstract

**Objective:**

*CLCN7* mutation caused abnormal osteoclasts, resulting in osteopetrosis. Depending on the type of mutation, *CLCN7* mutations can lead to severe or relatively benign forms of osteopetrosis. However, the serum metabolic alterations in osteopetrosis caused by *CLCN7* mutation are still unknown. We aimed to investigate the differences in the metabolome of osteopetrosis patients caused by *CLCN7* mutation versus healthy controls (HC), uncovering potential subtype diagnosis biomarkers.

**Methods:**

19 osteopetrosis patients caused by *CLCN7* mutation and 19 HC were recruited for liquid chromatography–tandem mass spectrometry analysis. The screened pathway was validated in the myeloid cell specific *Clcn7^G763R^* mutant mouse model by quantitative real-time PCR analysis.

**Results:**

Three metabolic pathways were significantly enriched, including glycerophospholipid metabolism (*P*=0.036948), arachidonic acid metabolism (*P*=0.0058585) and linoleic acid metabolism (*P*=0.032035). Ten differential expressed metabolites were located in these three pathways and classified ability with areas under the curve over 0.7 in receiver operating characteristic analysis, suggesting a certain accuracy for being the potential biological markers. Especially, we found that the proteins in glycerophospholipid metabolism were predicted to interact with ClC-7 and further verified that the expression of coding genes were significantly up-regulated in myeloid cell specific *Clcn7^G763R^* mutant mouse.

**Conclusion:**

This study provides data on serum metabolomics in osteopetrosis caused by *CLCN7* mutation and provides new potential metabolic markers and pathways for diagnosis and pathogenesis of osteopetrosis.

## 1 Introduction

Osteopetrosis is a rare metabolic bone disease characterized by abnormally increased bone mineral density that leads to bone marrow failure, compressive neuropathy, and skeletal dysmorphism (1). According to genetic patterns, it can be divided into autosomal dominant osteopetrosis (ADO), autosomal recessive osteopetrosis (ARO), and X-linked osteopetrosis (XLO) (1–3). ADO is the most common form of osteopetrosis, which incidence is estimated at 1:20,000 (4). Early on, ADO was thought to include two phenotypes, ADO I (OMIM 607634) and ADO II (OMIM 166600) (2). ADO I is characterized by a mutation in the LDL receptor related protein 5 (*LRP5*) gene that leads to high bone mass but does not result in fractures (5). ADO II is caused by impaired osteoclastic bone resorption that usually results from heterozygous missense mutations in the chloride channel 7 (*CLCN7*) gene (6, 7).

*CLCN7* is the gene that can not only cause severe recessive form of osteopetrosis, namely ARO, but also relatively benign form of osteopetrosis, namely ADO, depending on the type of *CLCN7* mutation (8). Furthermore, ADO II caused by *CLCN7* mutation accounts for 70% of ADO, which was the most common type of osteopetrosis (9). Therefore, this study focused on osteopetrosis caused by *CLCN7* mutations. *CLCN7* encodes the Cl^-^/H^+^ exchange transporter 7, also known as ClC-7, which generally localizes to the lysosomal compartment and the ruffled membrane of bone-resorbing osteoclasts (10). *CLCN7* mutations cause the abnormal osteoclasts cannot secrete acid and thus cannot dissolve the bone, resulting in osteopetrosis. This disease displays heterogeneity, with phenotypes exhibiting various levels of severity, spanning from asymptomatic to life-threatening (11–13). In the absence of genetic testing or typical radiographic findings, elevated levels of lactate dehydrogenase (LDH), aspartate aminotransferase (AST), and creatine kinase BB isozymes (CK-BB) are associated with osteopetrosis caused by *CLCN7* mutations (14, 15). Nevertheless, levels of these biomarkers have not been shown to correlate with disease severity, and normal values do not exclude the presence of mutations in the *CLCN7* gene (4). Therefore, there is an urgent need to find more specific and sensitive biomarkers.

There are many genetic studies on osteopetrosis, but currently, no serum metabolic research has been found on osteopetrosis caused by *CLCN7* mutations. By illuminating specific characteristics that differentiate between health and disease phenotypes, metabolome has become the cornerstone of understanding the differences between physiological and pathological processes, potentially allowing us to search for biomarkers with better sensitivity and specificity and new insights for researching osteopetrosis. Herein, we utilized the liquid chromatography–tandem mass spectrometry (LC-MS/MS) analysis to investigate the metabolic differences between healthy controls (HC) and osteopetrosis patients caused by *CLCN7* mutation, help decipher the primary metabolic pathway(s) that were altered in osteopetrosis and provide potential more specific and sensitive biomarkers.

## 2 Methods

### 2.1 Participants

Totally, 19 osteopetrosis patients caused by *CLCN7* mutation and 19 HC were enrolled in this study. Osteopetrosis patients were diagnosed by laboratory examination, imaging examination and gene testing. HC were recruited from the Health Examination Center. HC inclusion criteria were as follows (1) No metabolic or genetic bone diseases, such as hypoparathyroidism or hyperparathyroidism, hypothyroidism or hyperthyroidism, Paget’s disease, osteomalacia (2); No other medical history of liver, kidney, rheumatism, malignant tumor, etc. (3); No serious or active infection (4); Those who did not use any drugs for the treatment of metabolic bone disease or affecting bone metabolism. The descriptions of the participants are given in **Table 1**.

**Table 1:** Study population features.

		Osteopetrosis patients	Healthy controls	*P* value
Number of individuals		19	19	
Age, median (upper quartile, lower quartile)		33 (43, 18)	27 (31, 25)	0.279
Gender	Male, n (%)	4 (21.053%)	8 (42.105%)	0.163
	Female, n (%)	15 (78.947%)	11 (57.895%)	

All subjects or their guardians (for participants who were under 18 years old) provided informed consents and this study was approved by the Ethics Committee of Sixth People’s Hospital Affiliated to Shanghai Jiao Tong University School of Medicine.

### 2.2 Biospecimen collection and processing

38 fasting venous blood samples were collected and centrifuged at 3000 rpm for 15 min at 4°C. The supernatants were collected as serum samples. The quality control (QC) sample was prepared by mixing an equal aliquot of the supernatants from all of the serum samples.

### 2.3 LC-MS/MS analysis

LC-MS/MS analyses were performed using an UHPLC system (Vanquish, Thermo Fisher Scientific) with a UPLC BEH Amide column (2.1 mm × 100 mm, 1.7 μm) coupled to Orbitrap Exploris 120 mass spectrometer (Orbitrap MS, Thermo). The mobile phase consisted of 25mmol/L ammonium acetate and 25 ammonia hydroxide in water (pH = 9.75) (A) and acetonitrile (B). The Orbitrap Exploris 120 mass spectrometer was used for its ability to acquire MS/MS spectra on information-dependent acquisition mode in the control of the acquisition software (Xcalibur, Thermo).

### 2.4 Raw data preprocessing and statistical analysis

For clinical parameters, statistical analyses were using IBM SPSS Statistics version 25.0 software (IBM, Armonk, NY, USA). Normally distributed variables, skewed variables, and categorical variables were described using mean ± standard deviation, median (upper quartile, lower quartile), and frequency (percentage), respectively. Between-group comparisons of age between the osteopetrosis group and the HC group were made using Mann-Whitney U test, and of sex were made using chi-square test.

The raw data were processed with relative standard deviation de-noising. Then, the missing values were filled up by half of the minimum value. Also, internal standard normalization method was employed in this data analysis. The final dataset was scaled and logarithmic transformed to minimize the impact of both noise and high variance of the variables using SIMCA (V16.0.2, Sartorius Stedim Data Analytics AB, Umea, Sweden). After these normalizations, an unsupervised principal component analysis (PCA) and a supervised model of orthogonal projections to latent structures discriminate analysis (OPLS-DA) were performed. Then, OPLS-DA permutation test was used to test the validity of the model, using R (ggplot2, V 3.3.5). Metabolites which meet the *P*<0.05 (Student’s t-test) and the OPLS-DA model’s variable importance in the projection (VIP)>1 are considered as differentially expressed metabolites (DEMs). We visualize the results of DEMs in the form of volcano plots, using R (ggplot2, V 3.3.5). For each set of comparisons, we calculated the Euclidean distance matrix for the quantitative values of DEMs and presented them in a heatmap, using R (pheatmap, V 1.0.12).

Then, we annotated all DEMs through the Kyoto Encyclopedia of Genes and Genomes (KEGG) Pathway Database (http://www.kegg.jp/kegg/pathway.html), using R (base, V3.6.3), and calculated enrichment factor (the ratio of the number of DEMs annotated in a pathway to the number of all metabolites in that pathway, higher values indicate greater enrichment) by enrichment analysis with *P*-value (Fisher’s test), using R (ggplot2, V3.3.5). We analyzed the interactions between proteins in the key pathways enriched by DEMs and ClC-7 with the STRING database (http://string-db.org).

We also calculated the corresponding ratio for the quantitative values of DEMs, and took the logarithmic conversion at base 2. The receiver operating characteristic (ROC) curves were plotted for DEMs located in the differential key pathways, using R (plotROC V2.2.1, pROC V1.16.2), and the areas under the curve (AUC) were calculated to evaluate the accuracy of diagnosis model based on these DEMs.

### 2.5 RNA isolation and quantitative real-time PCR analysis

Total RNA was isolated from the bone tissues of both upper limbs from the myeloid cell specific *Clcn7^G763R^* mutant mouse model (16) according to the protocol supplied with TRIzol Reagent (Sigma, St. Louis, MO, USA). 1 ug total RNA was used for cDNA synthesis using the cDNA Synthesis Kit (Thermo Fisher Scientific, Waltham, MA, USA). Then quantitative real-time PCR analysis was performed (Roche LightCycler480II). All the primers were purchased from Beijing Tsingke Biotech Co., Ltd. The sequences of the primers were listed in **Supplementary Table S1**. mRNA expression levels were normalized to the respective β-actin mRNA and the 2^-ΔΔCt^ method was used to determine the relative amounts of mRNA transcribed. Independent samples t-test for normally distributed variables, Mann-Whitney U test for non-normally distributed variables.

## 3 Results

### 3.1 Clinical characteristics of enrolled subjects

The clinical features of 19 osteopetrosis patients with *CLCN7* mutation and 19 HC matched by age and sex are shown in **Table 1**. The results of Sanger sequencing in osteopetrosis patients with *CLCN7* heterozygous mutation are shown in **Table 2**.

**Table 2:** *CLCN7* mutation status in osteopetrosis patients.

Patient’s Number [Sex, Age (years old)]	Transcript	Mutation DNA level	Mutation protein level
1(Female, 14)	NM_001287.6	c.296A>G	p.Tyr99Cys
2(Female,40)	NM_001287.6	c.857G>A	p.Arg286Gln
3(Male,13)	NM_001287.6	c.857G>A	p.Arg286Gln
4(Male,32)	NM_001287.6	c.2299C>T	p.Arg767Trp
5 (Female,46)	NM_001287.6	c.2299C>T	p.Arg767Trp
6 (Female,11)	NM_001287.6	Heterozygous mutation in intron 20 near exon 20, resulting in putative aberrant splicing	
7 (Female,37)	NM_001287.6	c.1409C>T c.647_648dupTG	p.Pro470Leu p.Lys217X
8 (Female,18)	NM_001287.6	c.746C>T	p.Pro249Leu
9 (Female,33)	NM_001287.6	c.937G>A	p.Glu313Lys
10 (Female,33)	NM_001287.6	c.2284C>T	p.Arg762Trp
11 (Female,17)	NM_001287.6	c.856C>T	p.Arg286Trp
12 (Female,61)	NM_001287.6	c.2258C>G	p.Ser753Trp
13 (Male,26)	NM_001287.6	c.2377G>C	p.Gly793Arg
14 (Female,36)	NM_001287.6	c.647_648dupTG	p.Lys217X
15 (Male,27)	NM_001287.6	Heterozygous mutation in intron 24, resulting in A→G splicing mutation	
16 (Female,46)	NM_001287.6	c.2284C>T	p.Arg762Trp
17 (Female,42)	NM_001287.6	c.2236T>G	p.Tyr746Asp
18 (Female,43)	NM_001287.6	c.856C>T	p.Arg286Trp
19 (Female,50)	NM_001287.6	c.2299C>T	p.Arg767Trp

### 3.2 Multivariate data analysis of serum metabolites

The score scatter plot obtained by PCA of QC samples and experimental samples was shown in **Supplementary Figure S1**. As shown in the PCA score plot, all QC samples exhibited good overlap, indicating satisfactory stability in the analytical system, and the samples were essentially within the 95% confidence interval.

**Figure 1:**
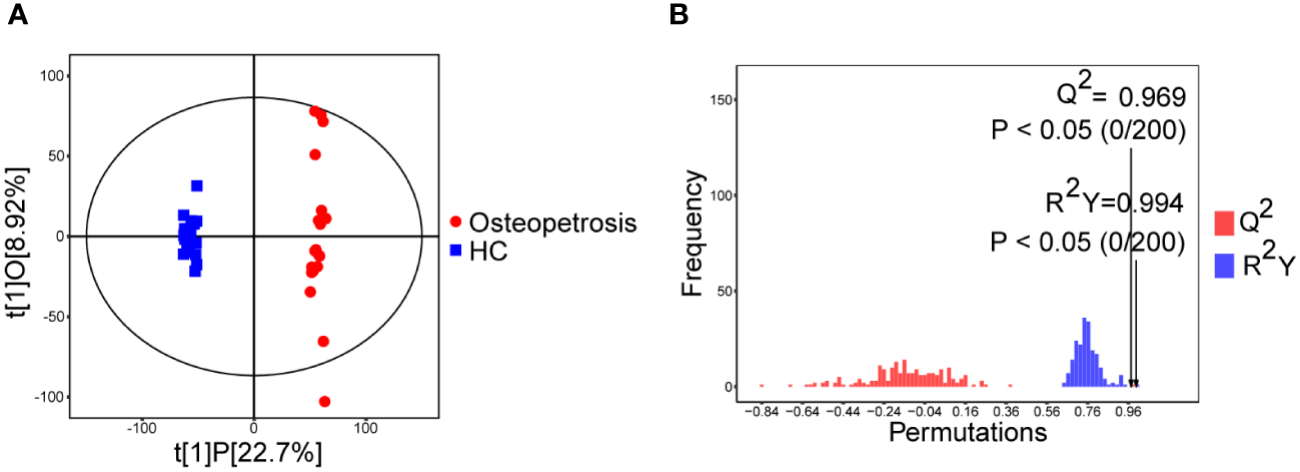
Multivariate analyses of serum metabolites. **(A)** Score scatter plot of OPLS-DA model; **(B)** Permutation plot test of OPLS-DA model. HC, healthy controls.

OPLS-DA analysis and OPLS-DA permutation test were used to characterize the differences between the two groups and evaluated the statistical significance of the model, as shown in **Figures 1A, B**. From the score scatter plot of OPLS-DA model, it can be seen that the osteopetrosis group and the HC group are very different on serum metabolites, and the within-group reproducibility of each group is good. The Q^2 ^= 0.969, R^2^Y=0.994 and *P*-values of Q^2^ and R^2^Y are less than 0.05, indicating that the OPLS-DA model was not overfitting and had high separating capacity.

### 3.3 Identification of the differential serum metabolites in the osteopetrosis group and HC group

**Figure 2:**
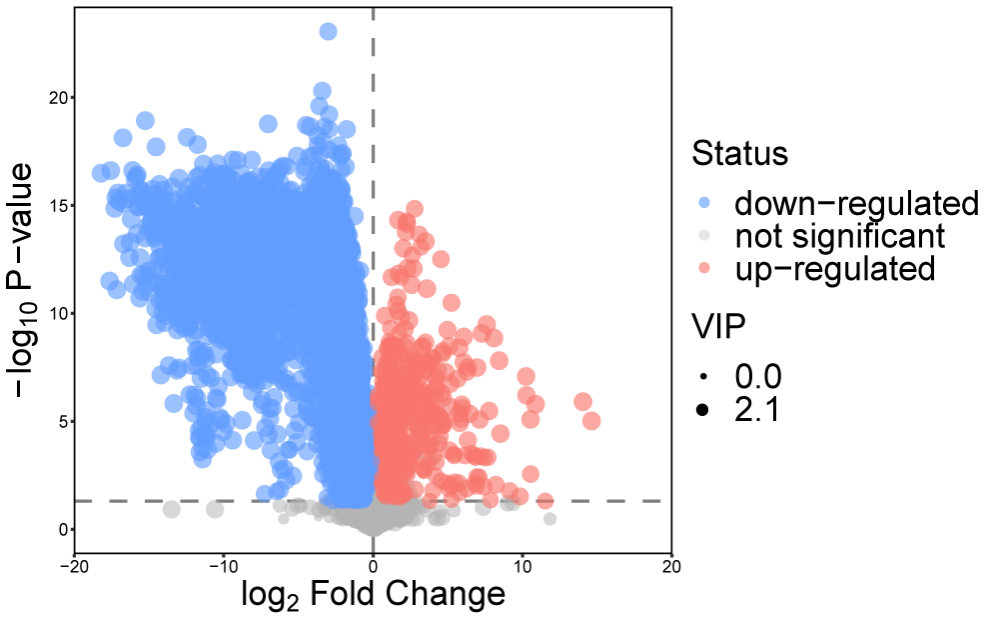
Volcano plot for differentially expressed metabolites for the osteopetrosis group and the healthy control group. VIP, variable importance in projection.

After the above analysis, combined with the results of univariate analysis and multivariate analysis, the metabolites between osteopetrosis patients and HC were screened. A total of 139 DEMs were screened based on the criteria of Student’s t-test *P*<0.05 and OPLS-DA model VIP>1, as shown in **Figure 2**. For each set of comparisons, we calculated the Euclidean distance matrix for the quantitative values of the DEMs, clustered them in a complete linkage method, and presented them in a heat map, showed in **Supplementary Figure S2** in **Supplementary materials**. As shown, there was a significant global difference in metabolic profiles between osteopetrosis patients and HC.

**Figure 3:**
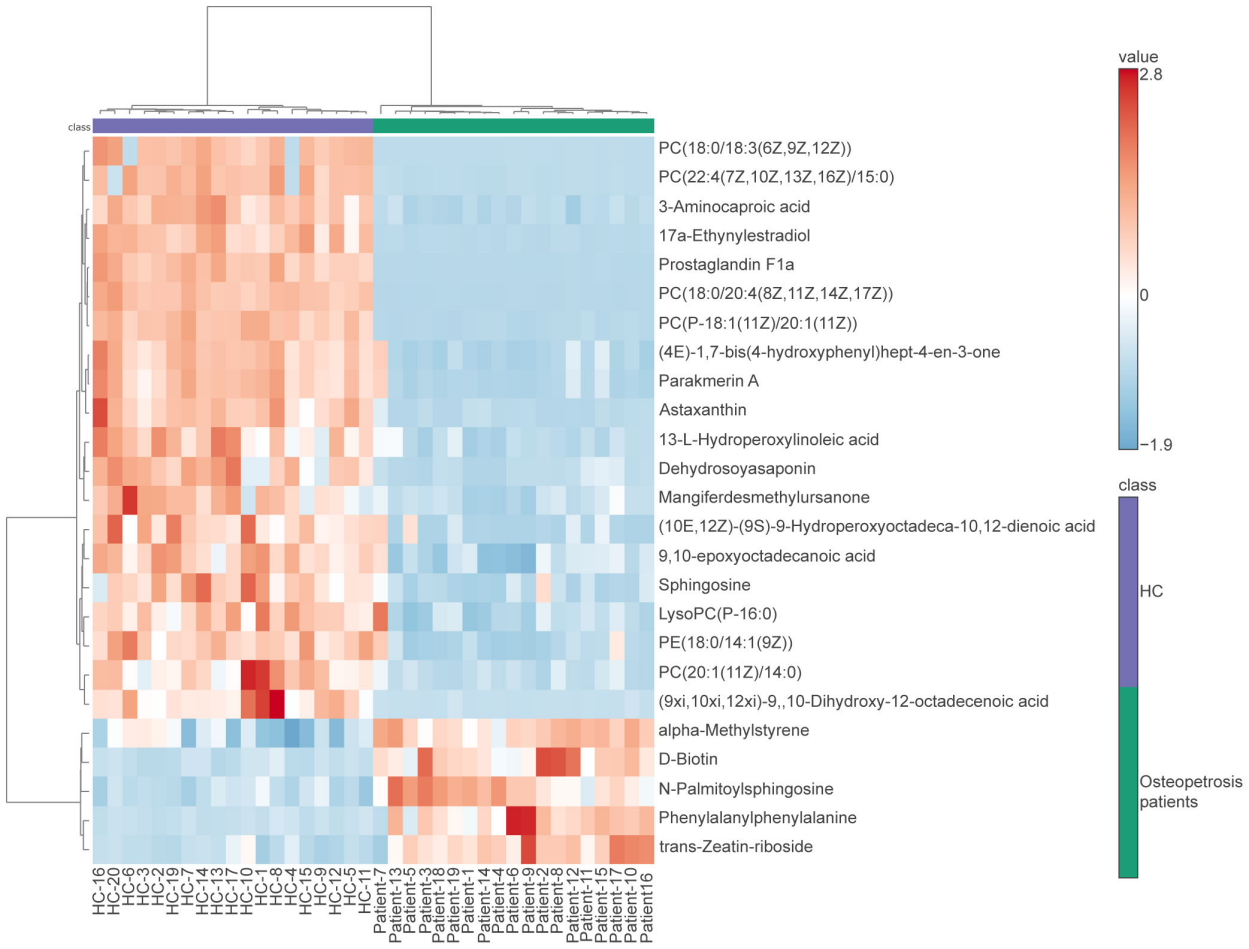
Heatmap of differentially expressed metabolites for subjects enrolled in this study. In the osteopetrosis group, patient 7 carried *CLCN7* biallelic pathogenic mutations and the rest of the patients carried *CLCN7* heterozygous mutation. HC, healthy controls.

Due to the specificity of *CLCN7* induced osteopetrosis, which means that biallelic *CLCN7* pathogenic mutations lead to ARO and heterozygous pathogenic mutations lead to ADO, we analyzed the concordance between biallelic pathogenic mutations and heterozygous mutation samples, as shown in **Figure 3** and **Table 2**. We actually found ARO patient (patient 7) was biallelic *CLCN7* mutations and had significant different metabolic levels from ADO patients caused by *CLCN7* heterozygous mutations. This suggests that the differential metabolism characteristics of individuals can not only distinguish osteopetrosis patients and HC, but also have potential ability to further discriminate different osteopetrosis subtypes induced by biallelic or heterozygous mutations of *CLCN7*.

### 3.4 Metabolic pathway analysis

According to the KEGG pathway database, all pathways mapped by DEMs were sorted out, and comprehensive analyses (including enrichment analysis and topology analysis) were used to find the key pathways with the highest correlation with metabolism differences between two groups. A total of 26 pathways were enriched, three of which were statistically significant, namely arachidonic acid (AA) metabolism (*P*=0.0058585), linoleic acid (LA) metabolism (*P*=0.032035), glycerophospholipid (GP) metabolism (*P*=0.036948), as shown in **Table 3**.

**Table 3:** KEGG pathway enrichment analysis of differentially expressed metabolites.

Pathway name	Hits Compounds	Total	Hits	Raw *P*	Holm *P*	FDR
Arachidonic acid metabolism	Prostaglandin G2; Delta-12-Prostaglandin J2; 15-KETE; Prostaglandin B2; 6-Ketoprostaglandin E1	62	5	0.0058585	0.46868	0.46868
Linoleic acid metabolism	13-oxo-octadecadienoic acid; 13-L-Hydroperoxylinoleic acid	15	2	0.032035	1	0.94411
Glycerophospholipid metabolism	Phosphorylcholine; Acetylcholine; Glycerophosphocholine	39	3	0.036948	1	0.94411

Pathway associated metabolite sets with raw P value<0.05 are shown in the table. Total, total number of metabolites in the metabolite pathway; Hits, number of DEMs in the metabolite pathway; Raw P, original P value in the enrichment analysis; Holm P, adjusted raw P value by Holm-Bonferroni method; FDR, false discovery rate.

For DEMs located in the screened three metabolic pathways, we evaluated their accuracy for the diagnosis of osteopetrosis using ROC analysis and calculating the AUC values, as shown in **Table 4**. All of them, a total of 10 DEMs meet AUC>0.7, which means they have a certain accuracy for being the potential biological markers for osteopetrosis with *CLCN7* mutation. In addition, there are 7 DEMs, Prostaglandin G2 (PGG2), Prostaglandin B2 (PGB2), 6-Ketoprostaglandin E1, 13-oxo-octadecadienoic acid (13-oxo-ODE), 13-L-Hydroperoxylinoleic acid (LOOH), acetylcholine and glycerophosphocholine, which satisfy AUC>0.9 and are of great value for diagnosis.

**Table 4:** Selected differentially expressed metabolites associated with osteopetrosis.

Name	Mode	Formula	VIP	*P*-Value	Log_2_FC	Expression	AUC	95%CI
Prostaglandin G2	NEG	C_20_H_32_O_6_	1.280	<0.001	-2.409	Down-regulated	0.909	0.815-1.000
Delta-12-Prostaglandin J2	NEG	C_20_H_30_O_4_	1.165	<0.001	-1.995	Down-regulated	0.892	0.780-1.000
15-KETE	NEG	C_20_H_30_O_3_	1.217	0.004	-1.242	Down-regulated	0.886	0.763-1.000
Prostaglandin B2	NEG	C_20_H_30_O_4_	1.341	<0.001	-1.580	Down-regulated	0.906	0.793-1.000
6-Ketoprostaglandin E1	NEG	C_20_H_32_O_6_	1.426	<0.001	-2.078	Down-regulated	0.931	0.848-1.000
13-oxo-octadecadienoic acid	NEG	C_18_H_30_O_3_	1.374	<0.001	-1.463	Down-regulated	0.903	0.808-0.999
13-L-Hydroperoxylinoleic acid	POS	C_18_H_32_O_4_	1.757	<0.001	-2.173	Down-regulated	0.992	0.973-1.000
Phosphorylcholine	POS	C_5_H_15_ClNO_4_P	1.102	0.002	-0.354	Down-regulated	0.812	0.674-0.950
Acetylcholine	POS	C_7_H_16_NO_2_^+^	1.562	<0.001	-2.419	Down-regulated	0.903	0.789-1.000
Glycerophosphocholine	POS	C_8_H_20_NO_6_P	1.502	<0.001	-1.106	Down-regulated	0.945	0.873-1.000

VIP, variable importance in projection; Log_2_FC, Log_2_(fold change); AUC, area under curve; 95%CI, 95% confidence interval; NEG, negative ion mode; POS, positive ion mode. All numbers retain three valid digits after the decimal point.

### 3.5 GP metabolism probably involved in the pathogenesis of osteopetrosis caused by *CLCN7* mutations

To better understand the physiological processes in which these ten DEMs are involved, we analyzed the top 25 metabolites with the strongest correlation to each of the ten DEMs, as shown in **Supplementary Figure S3**. Subsequently, for the 3 DEMs in the GP metabolism, the most relevant metabolites for each DEM, a total of 75, were included in the enrichment analysis, as shown in **Supplementary Table S2**. As can be seen in **Supplementary Table S2**, the raw *P* values of both the LA metabolism and the AA metabolism were less than 0.05, suggesting that the three pathways are closely linked.

**Figure 4:**
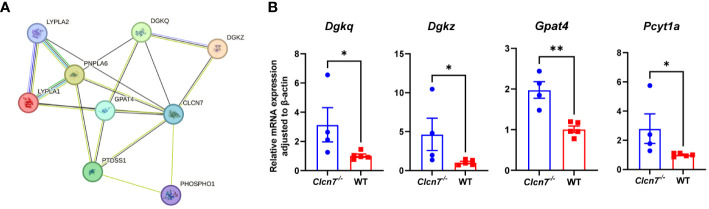
Validation of differential metabolic pathways. **(A)** proteins predicted to interact with ClC-7 in glycerophospholipid metabolism through the STRING database (version 12.0) with minimum required interaction score (0.15); **(B)** quantitative real-time PCR analysis results in the myeloid cell specific *Clcn7^G763R^* mutant mouse model. *P<0.05; **P<0.01; *Clcn7^-/-^*, *Clcn7^fl/fl^*; LysM-Cre^cre/-^; WT, *Clcn7^fl/fl^*; LysM-Cre^-/-^.

To further understand the role of the screened three pathways in osteopetrosis caused by *CLCN7* mutation, we analyzed the interactions of proteins in the three pathways with ClC-7 through the STRING database. Only proteins in GP metabolism are predicted to interact with ClC-7, which increases our interest in the role of this pathway in the pathogenesis of osteopetrosis caused by *CLCN7* mutation. As shown in **Figure 4A**, proteins predicted to interact with ClC-7 include diacylglycerol kinase theta (DGKQ), diacylglycerol kinase zeta (DGKZ), phosphatidylserine synthase 1 (PTDSS1), phosphoethanolamine/phosphocholine phosphatase 1 (PHOSPHO1), glycerol-3-phosphate acyltransferase 4 (GPAT4), lysophospholipase 1 (LYPLA1), lysophospholipase 2 (LYPLA2), and patatin like phospholipase domain containing 6 (PNPLA6). In the above proteins, PHOSPHO1 is the enzyme for the degradation of DEM phosphorylcholine, and LYPLA1 and LYPLA2 are the enzymes for the production of DEM glycerophosphocholine. In addition, we found that the production and degradation of DEMs located in GP metabolism were equally dependent on enzymes through the KEGG pathway database, including choline-phosphate cytidylyltransferase 1A and 1B (PCYT1A and PCYT1B; enzymes for the degradation of phosphorylcholine), choline O-acetyltransferase (CHAT; enzyme for the production of acetylcholine), acetylcholinesterase (ACHE; enzyme for the degradation of acetylcholine) and glycerophosphocholine phosphodiesterase 1 (GPCPD1; enzyme for the degradation of glycerophosphocholine).

To further investigate the role of the GP pathway in the pathogenesis of *CLCN7* mutations, we validated the expression of the genes encoding the above proteins in the myeloid cell specific *Clcn7^G763R^* mutant mouse model by quantitative real-time PCR analysis (**Figure 4B**). As shown in **Figure 4B**, the expression of *Dgkq* (*P*=0.027), *Dgkz* (*P*=0.027), *Gpat4* (*P*=0.002) and *Pcyt1a* (*P*=0.014) were significantly higher in *Clcn7^-/-^* (*Clcn7^fl/fl^*; LysM-Cre^cre/-^) mice than in the litter mate control (WT; *Clcn7^fl/fl^*; LysM-Cre^-/-^).

## 4 Discussion

This study presented a comprehensive metabolomics evaluation for osteopetrosis caused by *CLCN7* mutation in Chinese populations. We investigated the associations of metabolites with osteopetrosis to find specific and sensitive biomarkers and provide insights of osteopetrosis pathogenesis. To the best of our knowledge, this is the first study to analyze serum metabolic characteristics of osteopetrosis patients with *CLCN7* mutation by LC-MS/MS. In this study, we found that 10 metabolites including PGG2, Delta-12-prostaglandin J2 (D12-PGJ2), 15-KETE, PGB2, 6-Ketoprostaglandin E1, 13-oxo-ODE, LOOH, phosphorylcholine, acetylcholine and glycerophosphocholine, were significantly down-regulated in osteopetrosis patients caused by *CLCN7* mutation. Besides, we found GP metabolism was significantly enriched and probably involved in the pathogenesis of osteopetrosis.

GP metabolism, especially phosphorylcholine, acetylcholine, and glycerophosphocholine, were remarkably different between the two groups. GP is one of the important components of the bilayer membrane structure of the cell membrane, and play a vital role in cellular functions, including the regulation of transport processes, protein function and signal transduction (17–19). In previous studies, acetylcholine and glycerophosphocholine were shown to be associated with bone metabolism (20–22). Recent study has shown that ACHE as a hydrolytic enzyme will decrease acetylcholine concentrations, suppressing bone formation and increasing bone resorption (20). And Lips et al. confirmed that mice with gene deficiency of muscarinic acetylcholine receptor M3 had a declined trabecular bone volume, bone surface, and a higher trabecular pattern factor compared to wild type (21). This also means that acetylcholine may play an important role in bone metabolism. Øyen et al. have reported that subjects with low dietary total glycerophosphocholine are at higher risk of lower femoral neck bone mineral density compared to subjects with high levels, especially middle-aged men and elderly women (22).

Our group has previously reported 6 novel mutations of *CLCN7*, including G741R, a more severe mutation, with multiple fractures occurring in the patient carrying this mutation (23). It is now widely accepted that the pathogenesis of *CLCN7* mutations is due to the disruption of the acid environment necessary to dissolve the mineral component of bone, which prevents osteoclasts from functioning and leads to osteopetrosis (24). We therefore constructed the myeloid cell specific *Clcn7^G763R^* mutant mouse model to better explore the pathogenesis of *CLCN7*. And *Clcn7^-/-^* mice (*Clcn7^fl/fl^*; LysM-Cre^cre/-^) do have significantly reduced body weight, shorter femur length, and increased bone mineral density than the litter mate control (WT; *Clcn7^fl/fl^*; LysM-Cre^-/-^) (16). In this study, we found that the expression of *Pcyt1a* encoding the degradation enzyme of phosphorylcholine is statistically increased in *Clcn7 ^-/-^* mice compared to WT. We therefore hypothesize that there was the same increased expression of the *PCYT1A* gene in osteopetrosis patients caused by *CLCN7* mutation, which might account for the decrease level of phosphorylcholine. In addition, we identified some of the proteins in GP metabolism that may interact with ClC-7 through the STRING database, and subsequently verified the expression of genes encoding these proteins in *Clcn7^-/-^* mice. We found that the expressions of *Dgkq*, *Dgkz* and *Gpat4* were upregulated in *Clcn7^-/-^* mice. *Dgkq* and *Dgkz* encode DGKQ and DGKZ proteins, respectively, which are isozymes of diacylglycerol kinases (DGK) that convert diacylglycerol (DAG) to phosphatidic acid (25). Both DGKQ and DGKZ are expressed in bone residing cells, such as osteoclasts (26). Recent studies have shown that *Dgkz^-/-^* mice have increased osteoclast number in the trabecular and cortical bone and an osteoporotic bone phenotype due to the accumulation of DAG contributing to upregulation of c-Fos (26, 27). Therefore, we speculate that DGKQ and DGKZ may be involved in the pathogenesis of osteopetrosis by regulating the level of DAG. Taken together, GP metabolism probably plays an important role in the pathogenesis of osteopetrosis caused by *CLCN7* mutation.

Some limitations should be considered when interpreting our study. First, as osteopetrosis is a rare disease, the sample size of this study is inevitably small. Further studies with larger sample sizes are needed, especially to increase the number of biallelic pathogenic mutations to better analyze metabolic differences between biallelic pathogenic mutations, heterozygous mutation, and normal individuals. Second, the present statistical method of metabolomics cannot exclude other confounding factors which could influence osteopetrosis metabolism, such as age and gender. However, we balanced the age and gender between the two groups, which may reduce this impact to some extent. Third, due to the long-term sample collection in this study and the fact that the HC samples were collected from Health Examination Center, some biochemical test results were missing or incomplete.

To our knowledge, we demonstrated the serum metabolic profile of osteopetrosis caused by *CLCN7* mutation for the first time. In this study, a total of ten DEMs were found that can be used as potential diagnostic biological markers. Our study also highlighted that metabolic disorder in GP metabolism probably involved in the pathogenesis of osteopetrosis caused by *CLCN7* mutation. Overall, our study underscores the value of metabolomics in exploring biomarkers and biological mechanisms of osteopetrosis.

## Data availability statement

The original contributions presented in the study are publicly available. This data can be found here: MetaboLights database (www.ebi.ac.uk/metabolights/MTBLS11383).

## Ethics statement

The studies involving humans were approved by the Ethics Committee of Sixth People’s Hospital Affiliated to Shanghai Jiao Tong University School of Medicine. The studies were conducted in accordance with the local legislation and institutional requirements. Written informed consent for participation in this study was provided by the participants’ legal guardians/next of kin. The animal study was approved by Shanghai Sixth People’s Hospital Animal Care and Use Committee. The study was conducted in accordance with the local legislation and institutional requirements.

## Author contributions

XC: Writing – review & editing, Writing – original draft, Project administration, Methodology, Formal analysis, Conceptualization. ZiW: Writing – review & editing, Data curation. WF: Writing – review & editing, Data curation. ZhW: Writing – review & editing, Data curation. JG: Writing – review & editing, Methodology. CW: Writing – review & editing, Methodology. ZZ: Writing – review & editing, Supervision. XY: Writing – review & editing, Supervision. WH: Writing – review & editing, Supervision.
