# Supplementary material for: Metabolomics study of osteopetrosis caused by CLCN7 mutation reveals novel pathway and potential biomarkers
Source: Front Endocrinol (Lausanne). 2025 Feb 13;15:1418932. doi: 10.3389/fendo.2024.1418932 (PMC11865745; doi:10.3389/fendo.2024.1418932)
Supplement: Supplementary file 1 [file DataSheet_1.docx]

Supplementary Material

# Supplementary Figures and Tables

## Supplementary Figures


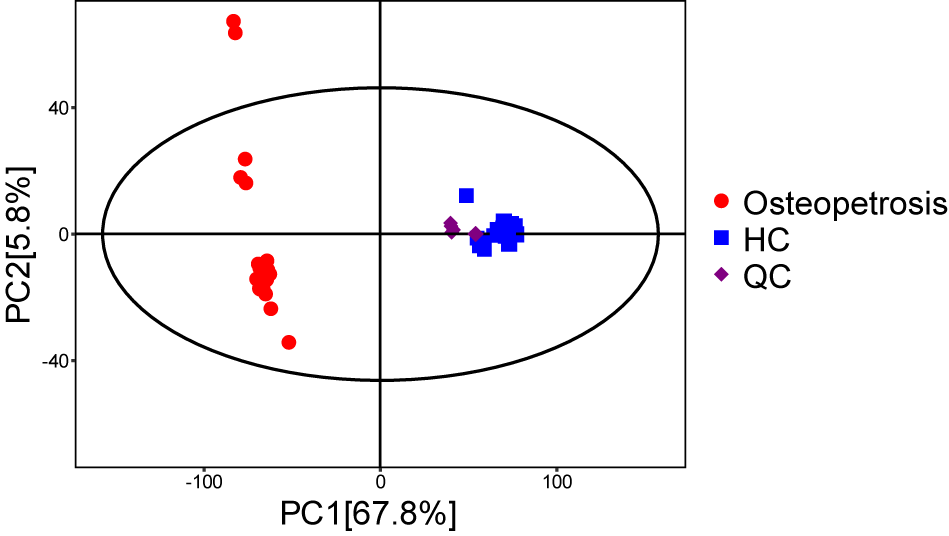


**Supplementary Figure 1.** PCA score scatter plot for all samples (including QC samples). HC, healthy controls; QC, quality control.


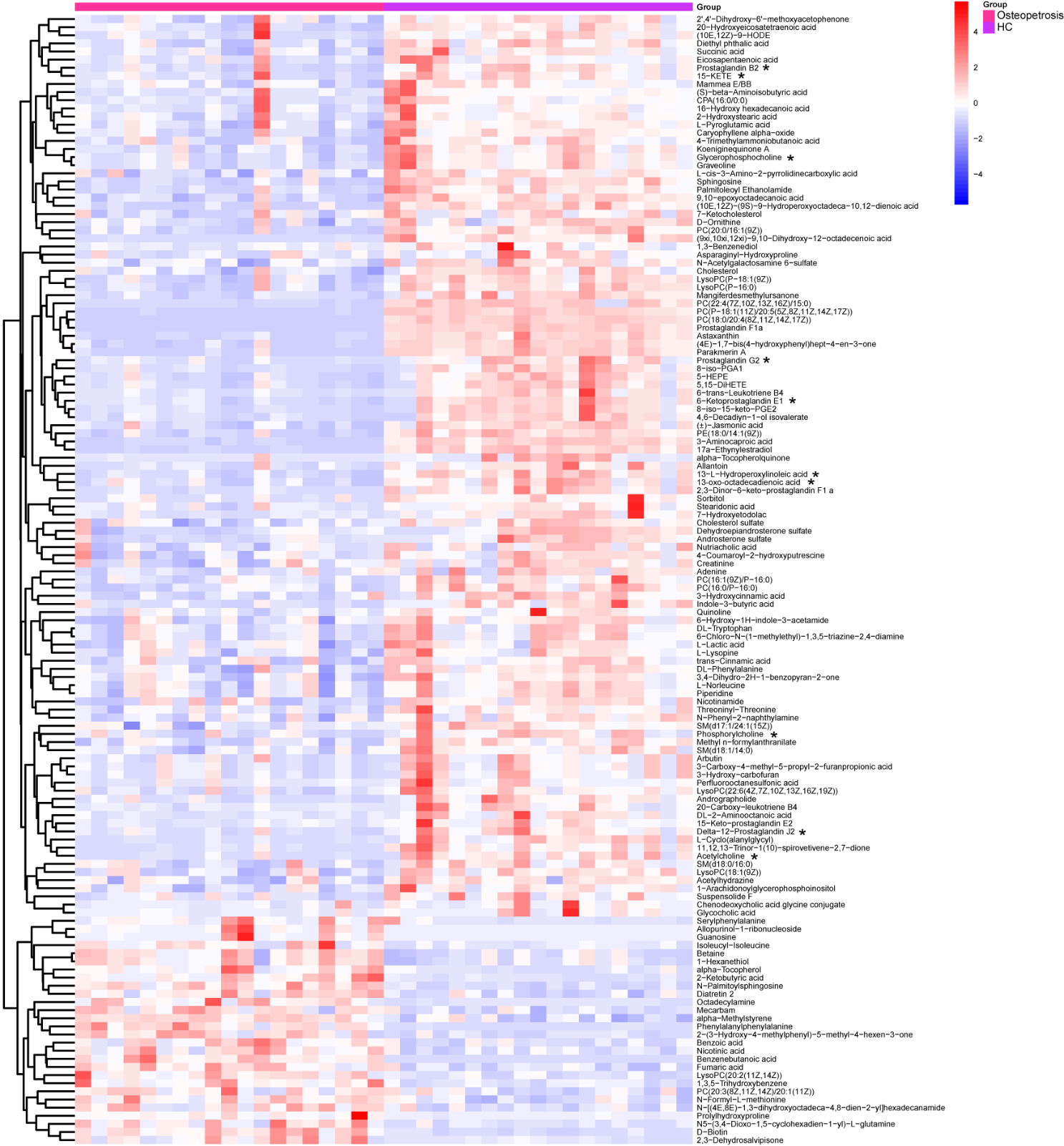


**Supplementary Figure 2.** Heatmap of differentially expressed metabolites for the osteopetrosis group and the healthy control group. Differentially expressed metabolites located in the screened metabolic pathways was labeled by *. HC, healthy controls.


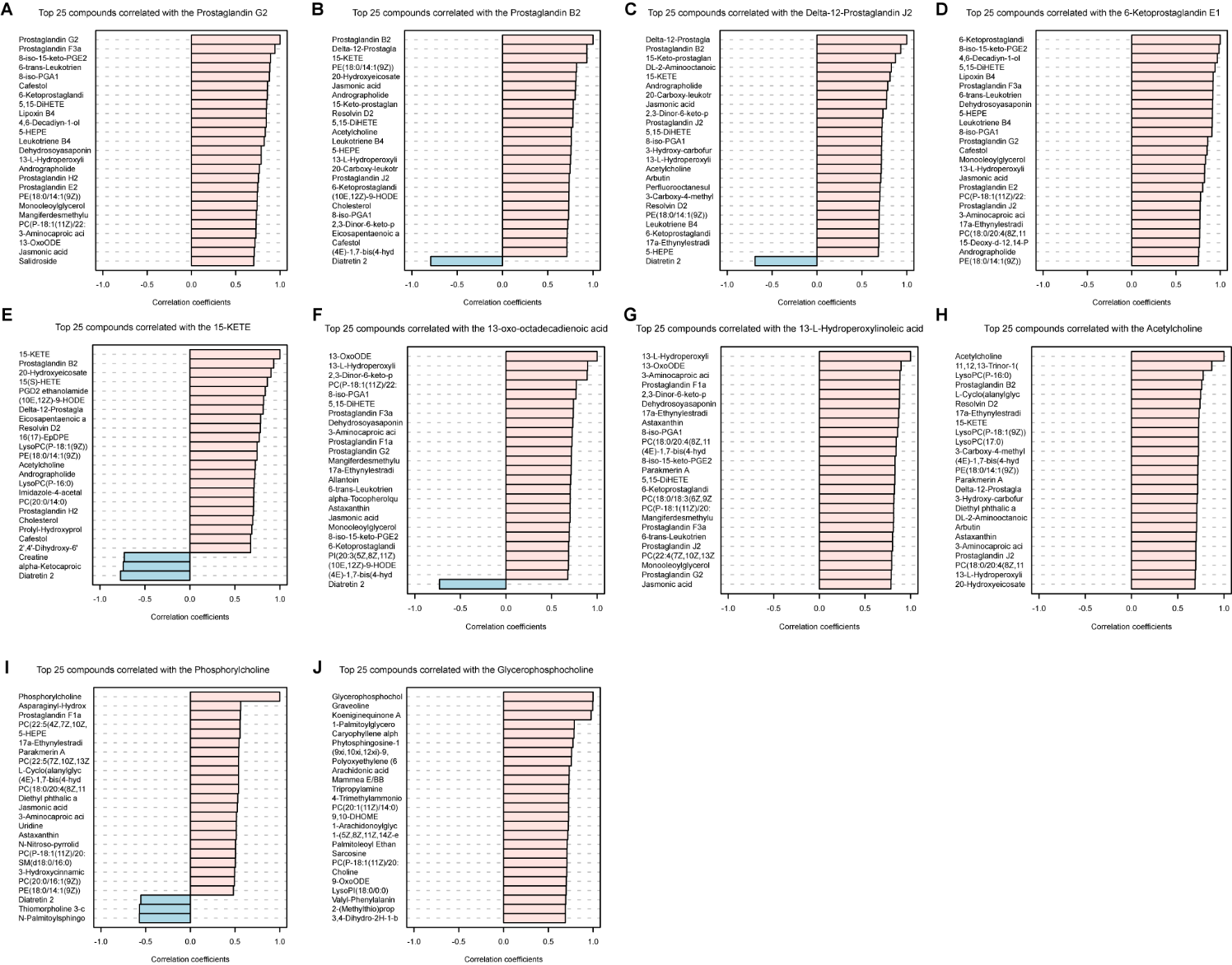


**Supplementary Figure 3.** Top 25 metabolites with the strongest correlation to differentially expressed metabolites located in the arachidonic acid metabolism, linoleic acid metabolism, glycerophospholipid metabolism. A to J are the top 25 compounds correlated with prostaglandin G2, prostaglandin B2, Delta-12-Prostaglandin J2, 6-Ketoprostaglandin E1, 15-KETE, 13-oxo-octadecadienoic acid, 13-L-Hydroperoxylinoleic acid, acetylcholine, phosphorylcholine and glycerophosphocholine, respectively.

## Supplementary Tables

**Table S1. Primer sequences used in the study**

| Gene  (mouse) | Forward Primer | Reverse Primer |
| --- | --- | --- |
| *Dgkq* | AGGTTTGAGAAGCCACGCATA | GACACGGAAGTAGGAGCCCT |
| *Dgkz* | CTCTTTGGGCACAGGAAAGC | TGCTGACTCACTCCAGTCCA |
| *Ptdss1* | GCAGGACTCTGAGCAAGGATG | GGCGAAGTACATGAGGCTGAT |
| *Phospho1* | CTGTACCGGCTGCATAACCTC | GTGTAGGGTTGCGTTGGCTGA |
| *Pcyt1a* | GATGCACAGAGTTCAGCTAAAGT | TGGCTGCCGTAAACCAACTG |
| *Pcyt1b* | TTCTCACGCAAGACCCTGAC | GCAAACTCCTACCAACAAGTAGC |
| *Chat* | GGCCATTGTGAAGCGGTTTG | GCCAGGCGGTTGTTTAGATACA |
| *Ache* | CTCCCTGGTATCCCCTGCATA | GGATGCCCAGAAAAGCTGAGA |
| *Lypla1* | TTTTCCTTCACGGATTGGGAG | GGGGACTTTTGATACCTGCAA |
| *Lypla2* | ATGTGTGGTAACACCATGTCTG | ACTCAGCCCCATCAGGTCAA |
| *Gpcpd1* | ATGACACCTTCTCAGGTCACT | GCTTTCCACAACACACTGTCTC |
| *Gpat4* | AGCTTGATTGTCAACCTCCTG | CCGTTGGTGTAGGGCTTGT |
| *Pnpla6* | CGGGTGCAGAAAACTCCAG | CGCATAATCTTCCGGCCATAGA |

**Table S2. KEGG pathway enrichment analysis of 75 metabolites most associated with the 3 differentially expressed metabolites in the glycerophospholipid metabolism**

| Pathway name | Total | Hits | Raw *P* | Holm *P* | FDR |
| --- | --- | --- | --- | --- | --- |
| Glycerophospholipid metabolism | 36 | 4 | 6.78E-05 | 0.0054272 | 0.0054272 |
| Glycine, serine and threonine metabolism | 33 | 2 | 0.020811 | 1 | 0.71506 |
| Linoleic acid metabolism | 5 | 1 | 0.034479 | 1 | 0.71506 |
| Arachidonic acid metabolism | 44 | 2 | 0.035753 | 1 | 0.71506 |

Pathway associated metabolite sets with raw *P* value<0.05 are shown in the table. Total, total number of metabolites in the metabolite pathway; Hits, number of differentially expressed metabolites in the metabolite pathway; Raw *P*, original *P* value in the enrichment analysis; Holm *P*, adjusted raw *P* value by Holm-Bonferroni method; FDR, false discovery rate.
